# An experimental chimeric hepatitis E virus vaccine elicits both local and systemic immune responses

**DOI:** 10.3389/fmicb.2024.1512018

**Published:** 2024-12-24

**Authors:** Melisa Florencia Müller, Jacinto Sacur, Julia Matias Brancher, María Daniela Vera, Lorena Arce, María Fernanda Raya-Tonetti, Haruki Kitazawa, Julio Villena, María Guadalupe Vizoso-Pinto

**Affiliations:** ^1^Infection Biology Laboratory, Instituto Superior de Investigaciones Biológicas (INSIBIO), CONI-CET-UNT, Tucumán, Argentina; ^2^Laboratory of Immunobiotechnology, Reference Centre for Lactobacilli (CERELA-CONICET), Tucumán, Argentina; ^3^Food and Feed Immunology Group, Laboratory of Animal Food Function, Graduate School of Agricultural Science, Tohoku University, Sendai, Japan; ^4^Livestock Immunology Unit, International Education and Research Center for Food and Agricultural Immunology (CFAI), Graduate School of Agricultural Science, Tohoku University, Sendai, Japan; ^5^Laboratorio Central de Ciencias Básicas, Facultad de Medicina, Universidad Nacional de Tucumán, Tucumán, Argentina

**Keywords:** hepatitis, antigen-display, lactic acid bacteria, recombinant antigen, ORF2, immunomodulatory bacterium-like-particles

## Abstract

**Introduction:**

The development of a hepatitis E virus (HEV) vaccine is critical, with ORF2 capsid protein as the main target. We previously demonstrated that oral coadministration of recombinant ORF2 with immunomodulatory bacterium-like-particles (IBLP) induces a specific immune response in mice, particularly using IBLP derived from *Lacticaseibacillus rhamnosus* IBL027 (IBLP027), which was effective in eliciting a local humoral response. IBLP are non-live bacteria with adjuvant and carrier properties, serving as a platform for exposing proteins or antigens fused to LysM (lysine motif) domains, protein modules that bind to cell wall polysaccharides like peptidoglycan.

**Materials:**

We cloned the most immunogenic domain of ORF2 (O2P2) fused to five LysM domains (LysM_5_O2P2) and displayed this chimeric protein on the surface of IBLP027 to create a prototype vaccine (IBLP027-LysM_5_O2P2). We evaluated its capacity to induce an immune response *in vivo* by immunizing mice with three doses of either the experimental vaccine or the chimeric protein alone, using an oral or a combined schedule with subcutaneous priming followed by oral boosting. Control groups received IBLP027. Sera and small intestine fluid were analyzed for humoral response, while Peyer’s patches and spleen immune cells were used for ex vivo stimulation with capsid protein to assess cellular response.

**Results:**

The oral scheme failed to elicit an IgG response, but this was overcome by a subcutaneous priming dose followed by oral boosters, which led to increasing IgG titers in the combined scheme. The highest IgG titers were seen in the vaccine prototype group. Most groups produced significantly higher IgA levels in intestinal fluid, especially in those that received the oral scheme. Cellular response studies showed increased tumor necrosis factor (TNF)-α, interferon (IFN)-γ interleukin (IL)-4, and IL-17 levels in groups receiving the chimeric protein via oral or combined schedules.

**Conclusion:**

Further and continuous research is needed to better understand both the needs and expectations of students and supervisors in different academic realities, including in Veterinary Medicine schools, from which the information available on the subject is scarce.

## 1 Introduction

The hepatitis E virus (HEV) is a causative agent of hepatitis, responsible for 20 million infections and 44,000 deaths annually (Webb and Dalton, 2019; World Health Organization, 2023). HEV is a single-stranded, positive-sense RNA virus with a genome comprising three open reading frames (ORFs). ORF1 encodes a non-structural polyprotein necessary for HEV replication, ORF2 the capsid protein, and ORF3 a small phosphoprotein involved in virion release (Sayed and Meuleman, 2020; Wang et al., 2018). The main target for vaccine development is ORF2, which consists of three domains: S (shell, 129–319aa), M (middle, 320–455aa), and P (protruding, 456–606aa) (Yin and Feng, 2019). The P domain within ORF2 is crucial for viral-host interactions and contains neutralizing epitopes (Zhao et al., 2015; Cancela et al., 2022).

Among the genotypes that primarily infect human beings, HEV genotype 1 (HEV-1) and HEV-2 are mainly transmitted through the fecal-oral route, typically from consuming contaminated water (World Health Organization, 2023; Cancela et al., 2022). Additionally, cases of vertical transmission and transmission through blood transfusions have been documented (Sharma et al., 2017; Wu et al., 2020; Marion et al., 2019). HEV-3 and HEV-4 circulate in animals such as pigs, wild boars, and deer, but also infect humans. Swine serve as the main reservoir for HEV-3, and although the virus does not manifest clinically in swine, close contact with infected pigs or the consumption of meat derivatives increases the risks of infection. Reducing HEV infection rates in humans and swine is essential to limit zoonotic transmission and mitigate public health risks. By controlling the virus in swine, might be substantially reduced.

A recombinant vaccine, Hecolin^®^, based on the capsid protein ORF2 (368–606aa) of a Chinese HEV-1 strain, has been licensed in Pakistan and China (Zhang et al., 2016; Øverbø et al., 2023). This intramuscular vaccine has proven effective (Huang et al., 2024; Kao et al., 2024); therefore, the World Health Organization, during the latest SAGE (Strategic Advisory Group of Experts on Immunization) meeting, recommended vaccination for women of childbearing age, including pregnant women, during outbreaks (Strategic Advisory Group of Experts, 2024). In fact, the first ever mass vaccination campaign against hepatitis E in response to an outbreak was implemented in 2022 in South Sudan targeting 27,000 residents (Nesbitt et al., 2024). Nevertheless, the WHO also highlighted that, there is limited data regarding the vaccine safety in certain subpopulations such as pregnant women, pediatric subjects (<16 years of age) and individuals with underlying conditions such as immunosuppression (Strategic Advisory Group of Experts, 2024). Additionally, data on cross-protection against HEV-3 remain unavailable (World Health Organization, 2015). Recombinant vaccines are generally considered safe (Lenart et al., 2024). They have been successfully used for protection against several infectious diseases, such as those caused by Severe acute respiratory syndrome coronavirus 2 (SARS-CoV-2), hepatitis B virus (HBV) and human papilloma virus (HPV) (Lenart et al., 2024; Fabrizi et al., 2020; Zaman et al., 2024). Nevertheless, a key limitation of recombinant vaccines is their reliance on adjuvants to enhance the immune response.

Lactic acid bacteria (LAB) have long been studied as potential delivery systems and/or adjuvants for mucosal vaccines due to their immunomodulatory properties and GRAS (generally regarded as safe) status. Recombinant LAB expressing pathogen antigens on their cell walls have been used as oral or nasal vaccines with the ability to induce specific mucosal and systemic immune responses in animal models (Villena et al., 2008; Bermúdez-Humarán et al., 2004). However, the administration of genetically modified organisms raises several concerns including environmental, health, and ethical considerations.

An alternative to genetically modified LAB is bacterium-like particles (BLPs), produced by treating LAB with hot acid to kill the bacteria, remove DNA and cytoplasmic proteins, and expose cell wall peptidoglycan (Raya Tonetti et al., 2020). Some BLPs, depending on the strain and administration route, act as adjuvants, modulating mucosal immune responses (Raya Tonetti et al., 2020; Villena and Kitazawa, 2020; Sudo et al., 2023). Their cost-effectiveness and ease of production have made them useful in developing mucosal vaccine candidates (Wang et al., 2022; Li et al., 2019; Liu et al., 2017). Further, our prior findings indicated that oral immunization with recombinant ORF2, co-administered with BLPs derived from immunobiotic lactobacilli (IBLP), elicits a specific immune response (Arce et al., 2020).

As HEV primarily enters through mucosal surfaces, an effective mucosal vaccine should block viral propagation at the entry point. Designing such vaccines requires strategies to protect antigens from the harsh mucosal environment, such as anchoring recombinant proteins to the surface of beneficial microorganisms like LAB (Michon et al., 2016).

The LysM motif, a cell wall binding domain, is an ubiquitous motif, spanning 42–65 amino acids, found in over 4,000 proteins across prokaryotes and eukaryotes. These motifs selectively attach to N-acetylglucosamine residues of peptidoglycan through non-covalent interactions (Desvaux et al., 2006; Bosma et al., 2006). In previous studies, LysM domains have been fused to antigens allowing the antigen to be exposed at the BLP surface. In this way, the BLP acts both as adjuvant and carrier (Li et al., 2019; Liu et al., 2017; Bosma et al., 2006; Raya-Tonetti et al., 2021). This binding is immediate and robust, as we have evidenced it before (Raya-Tonetti et al., 2021).

**Figure 1:**
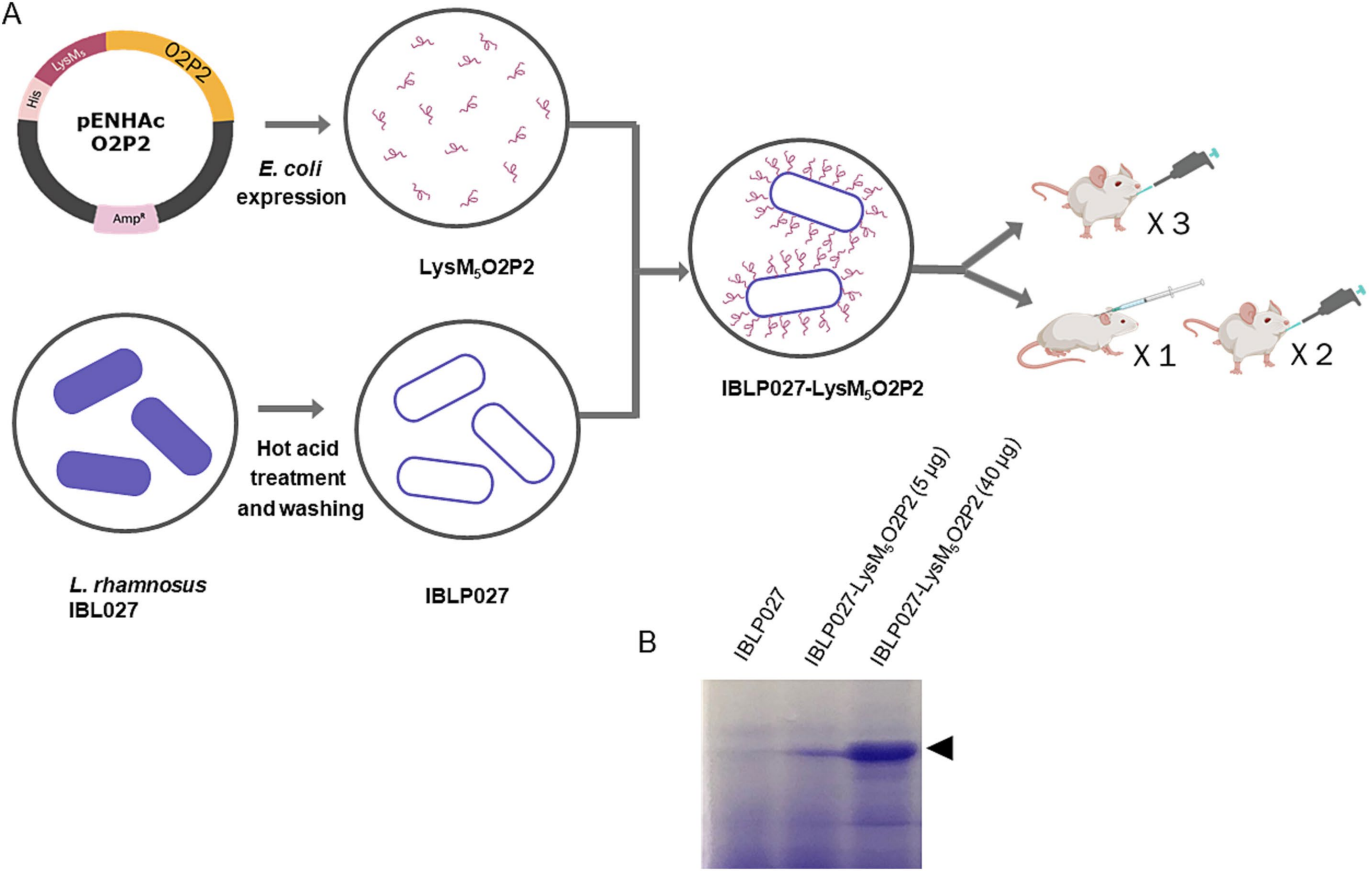
Schematic diagram of O2P2 protein, IBLP027 particle preparation and mice immunization. **(A)** pENHAcO2P2 is the expression plasmid used to generate the fusion protein. IBLP obtained by heat and acid treatment were mixed to obtained the LysM_5_O2P2-IBLP complexes for immunization of mice following either an oral or a combined schedule with subcutaneous + oral immunization of BALB/c mice. **(B)** The SDS-PAGE gel stained with Coomassie shows the IBLPs alone, and the complexes loaded with two different concentrations of LysM_5_O2P2, 5 and 40 μg, respectively.

The goal of this study was to clone the protruding domain of HEV-3 ORF2 (O2P2) as a chimeric protein fused to LysM motifs (LysM_5_O2P2) to expose it on the surface of *Lacticaseibacillus rhamnosus* IBL027 derived IBLPs. Then, to evaluate the immunogenic properties of LysM_5_O2P2 alone or IBLP027-LysM_5_O2P2 complexes by administering them to BALB/c mice following two different administration schedules: a three-dose oral regimen or a combined regimen involving one subcutaneous dose followed by two oral boosters (Figure 1A).

## 2 Materials and methods

### 2.1 Construction of recombinant plasmids

O2P2 with *att*B sites was amplified by a nested PCR using the pMKORF2 GT-3 plasmid, a designed plasmid, as template. Briefly, the “first round PCR primer set” used was HEVORF2-452-608-Fw: 5′-AAAAAGCAGGCTCCGCCATGCCTACCCCTTCCCCTGCT-3′ and HEVORF2-452-608-Rv: 5′-AGAAAGCTGGGTCAGCCAGAGCGGAGTGGGG-3′. The “second round PCR primer set” was Fw: 5′-GGGGACAAGTTTGTACAAAAAAGCAGGCT-3′ and Rv: 5′-GGGGACCACTTTGTACAAGAAAGCTGGGTC-3′. The PCR product was gel purified using the Agarose gel DNA extraction Kit 2 (Roche) and recombinantly cloned into containing the *att*P sites using the BP Clonase II enzyme mix (Invitrogen, Germany) according to the manufacturer’s instructions. BP reactions were incubated at room temperature overnight and subsequently transformed into chemically competent *E. coli* TOP10. Plasmidic DNA of individual colonies grown on LB plates supplemented with 12.5 μg/mL gentamicin (Invitrogen, Germany) was isolated using the QIAprep Spin Miniprep Kit (Qiagen, Germany) (Vizoso Pinto et al., 2010). The pENTRO2P2 vector was verified by *BanII* (New England Biolabs, Germany) restriction analysis and forward sequencing (Macrogen).

LR recombination reactions using the LR Clonase II enzyme mix (Invitrogen, Germany) were performed according to the manufacturer’s instructions. The pENTRO2P2 vector containing the O2P2 sequence flanked by *att*L sites was recombinatorially cloned into the customized vector pENHAc[rfB] containing the *att*R sites. This vector allows the expression of chimeric proteins with a LysM_5_ anchor (Raya-Tonetti et al., 2021). LR clonase reaction was incubated at 37°C for 2 h and subsequently transformed into chemically competent *E. coli* TOP10. Plasmid DNA of individual colonies grown on LB plates supplemented with 100 μg/mL ampicillin (Sigma-Aldrich, Germany) was isolated as described above. The integrity of the resulting pENHAcO2P2 vector was verified by *EcoRV* (New England Biolabs, Germany) (Vizoso Pinto et al., 2010).

### 2.2 Expression and purification of ORF2 and the chimeric protein LysM_5_O2P2

Chemical competent *E. coli* Rosetta cells were transformed by heat shock with pENHAcO2P2 or pETG-N-His-ORF2, and the positive clones were selected on plates with LB medium supplemented with 100 μg/mL ampicillin and 17 μg/mL chloramphenicol. Individual colonies were selected, and the production of the recombinant protein was evaluated. The LB broth with corresponding antibiotics was inoculated with each clone, after growing to A_600nm_ = 0.3 at 37°C with shaking, the protein expression was induced with 1 mM isopropyl-β-d-thiogalactoside (IPTG), until an A_600nm_ = 0.7–0.8 was reached. Bacterial lysis was performed as described by Raya-Tonetti et al. (2021). The chimeric protein LysM_5_O2P2 was mainly found in the inclusion bodies, which were solubilized in a buffer containing 8 M urea obtaining a fraction called Sn2. ORF2 was mainly found in cytoplasm and purified under native conditions by affinity chromatography (NiNTA, Thermo Scientific). This protein was used for the *ex vivo* stimulation assays and the ELISA assays. Protein expression was evaluated with SDS-PAGE followed by Coomassie Brilliant Blue’s staining and checked by western blotting using a mouse monoclonal anti-RGS-His antibody (Qiagen).

### 2.3 Preparation of IBLP027-LysM_5_O2P2 complexes

*L. rhamnosus* IBL027 deposited in the Culture Collection of the Faculty of Biochemistry, Chemistry and Pharmacy of the National University of Tucumán (Tucumán, Argentina), was grown for 12 h at 37°C in Man-Rogosa-Sharpe (MRS) broth (final log phase). IBLP were prepared as described before and stored at −20°C until use (Arce et al., 2020). For protein purification, IBLP particles were used to bind LysM_5_-tagged proteins. Initially, a binding assay was performed by mixing two volumes of the Sn2 fraction with one volume of 10^9^ IBLP particles per mL, incubating the mixture for 3 min at room temperature. The mixture was then centrifuged at 7,500 rpm for 3 min, and the supernatant was discarded. The resulting pellet, containing IBLP027-LysM_5_O2P2 complexes, was washed with PBS (pH 7.4), and resuspended in one volume of PBS. At this stage, the LysM-tagged proteins were strongly bound to the exposed peptidoglycan of the IBLP particles via non-covalent interactions. To eliminate non-specific binding of *E. coli* proteins, the chimeric protein was eluted in one volume of 8 M urea in PBS. Following centrifugation at 7,500 rpm for 3 min, three eluates enriched with the chimeric protein were collected. The protein concentration was determined using the Bradford method (Bio-Rad), following the manufacturer’s instructions, and the purified protein was stored at −70°C until use. The control protein, LysM_5_O2P2, was dialyzed before immunization. For obtaining the IBLP027-LysM_5_O2P2 complexes, the eluates, containing the purified protein, were diluted 1:100 in PBS and re-incubated with IBLP and were washed three times with PBS to get rid of urea traces. The final bound protein was assessed using SDS-PAGE, and band intensities were quantified using ImageJ by comparing them to an albumin standard curve. An in-house monocyte activation test was conducted to confirm that the products were free of endotoxins (Vipond et al., 2019).

### 2.4 Western blotting

To confirm the presence of the chimeric protein, the bacterial supernatant and the purified protein were subjected to SDS-PAGE, and subsequently transferred onto nitrocellulose using a Trans-Blot^®^ Semi-Dry Transfer Cell (BioRad) at 22 V for 45 min. Initially, the membrane was blocked with 5% skimmed milk in PBS-T, a mouse anti-RGS-His antibody (1:2,000, Qiagen) was applied. Subsequently, a second antibody, anti-mouse peroxidase-labeled (α-Mo Pox, 1:2,000, Dako), along with DAB as the substrate were used to reveal the specific band.

### 2.5 Experimental vaccines and mice immunization protocol

**Figure 2:**
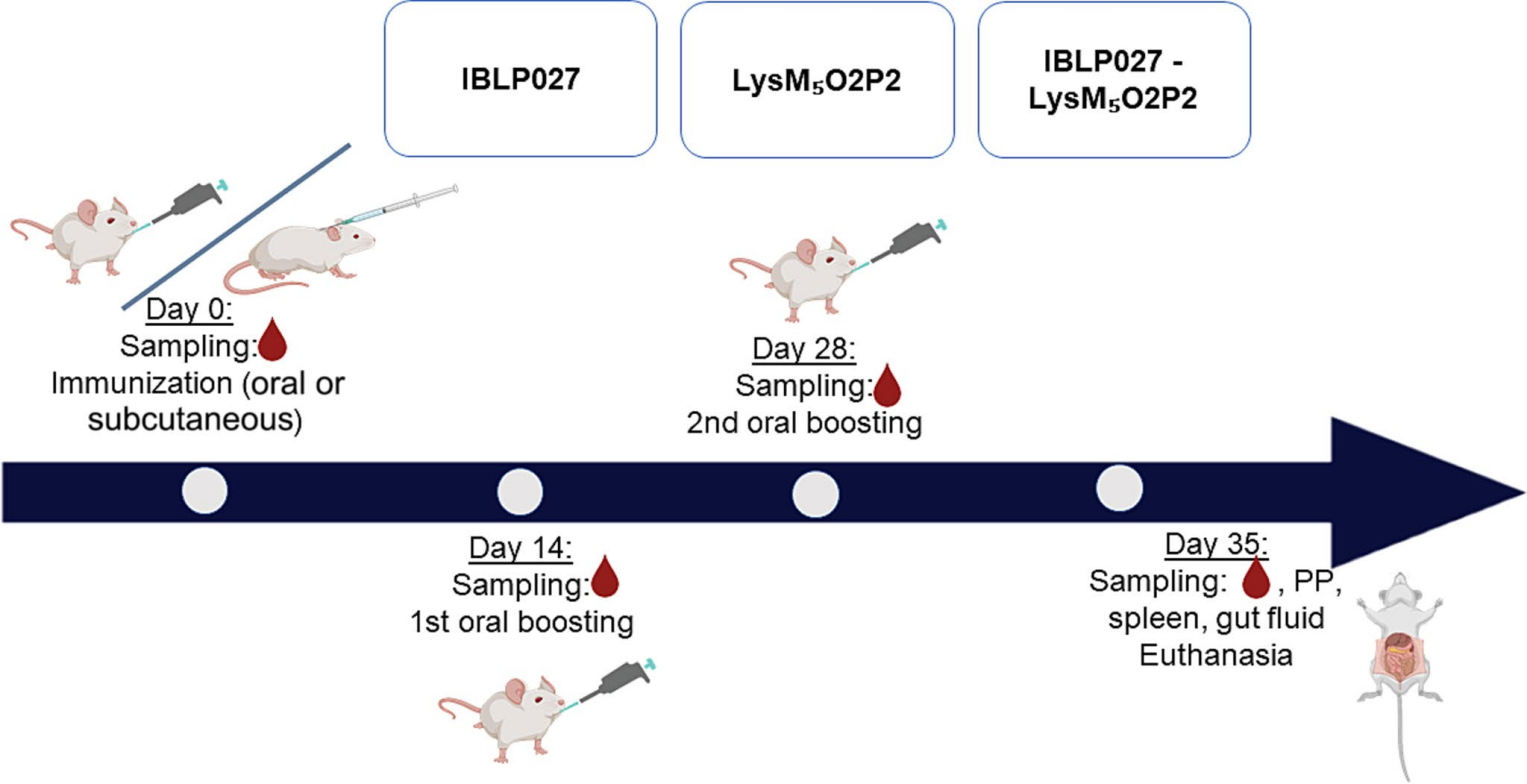
Immunization protocol: mice were divided into three groups (5 mice each) and subjected to two immunization schemes: three oral doses or one subcutaneous dose followed by two oral doses. Group 1 (IBLP027): control group receiving only IBLP027. Group 2 (LysM_5_O2P2): immunized with the chimeric protein LysM_5_O2P2 without adjuvant. Group 3 (IBLP027-LysM_5_O2P2): immunized with LysM_5_O2P2 displayed on the surface of IBLP027.

Forty μg of LysM_5_O2P2 alone or the same amount displayed on the surface of 2.5 × 10^8^ IBLP were administered to six-week-old male Balb/c mice obtained from a closed colony kept at the National University of Rio Cuarto (Cordoba, Argentina). During the experiments, animals were fed with conventional balanced diet *ad libitum*. Mice were randomly divided into groups (*n* = 5) to receive three immunizations every 14 days with either the chimeric protein or the complexes IBLP027-LysM_5_O2P2 following either an oral route or a combined schedule in which the first dose was administered subcutaneously followed by two oral boosts. Immunizations with IBLP027 alone served as a negative control (Figure 2).

### 2.6 Tissue and fluids sampling

Blood samples were collected from the submandibular capillary plexus immediately before each immunization (t0, t1, t2). Seven days after the last dose, mice were euthanized, blood was obtained through cardiac puncture (t3) and intestinal fluid was collected by flushing the small intestine with PBS supplemented with a protease inhibitor cocktail (Complete, Roche). Sera and fluids were frozen at −70°C until use.

Mononuclear cells from Peyer’s patches (PP) and spleen were isolated following standard methodologies. Briefly, PP or spleen cell suspensions were obtained by mechanical disaggregation. After lysing the erythrocytes by a 10 s incubation in distilled water and five washes in cold PBS, the cells were resuspended in PBS and quantified in a Neubauer chamber. Viability was determined by trypan blue exclusion.

### 2.7 Specific antibodies determination

Anti-HEV ORF2 specific antibodies in serum and intestinal fluid were determined using enzyme-linked immunosorbent assays (ELISA) as reported by Arce et al. (2020), with minor changes (Zhang et al., 2016). Purified native HEV GT3 ORF2 was coated (1 μg/mL) onto 96-well high-binding microtiter plates and blocked with 1% gelatine. Gut fluid (diluted 1:2) and serial serum dilutions were incubated for 1 h at 37°C. Anti-ORF2 specific mouse antibodies were detected using HRP-conjugated antibodies directed to mouse IgG-Fc (MP Biomedicals, Inc.) (1:1,000) or biotin-conjugated antibodies against IgA-Fc (1:250) (Sigma-Aldrich, St. Louis, MO, United States) followed by streptavidin-HRP conjugated (1:1,000) (Sigma-Aldrich, St. Louis, MO, United States). The endpoint enzymatic activity was detected with TMB (Sigma-Aldrich) as substrate. The reaction was stopped with 0.1 M H_3_PO_4_ and measured at 450 nm in a microplate reader. When possible, the specific IgG titer was obtained as GMT. The cutoff value was determined as the mean absorbance plus 3 standard deviations of a panel of sera from naïve mice (t0).

### 2.8 Cytokine measurement procedures

To investigate both local and systemic cellular immune responses, mononuclear cells obtained from PP and spleen were individually subjected to *ex vivo* stimulation with the HEV capsid protein (0.5 μg/well) in 24 well plates seeded with 4 × 10^6^ cells/well. Subsequently, the levels of tumor necrosis factor (TNF)-α, interferon (IFN)-γ, interleukin (IL)-4, and IL-17 were quantified from the culture supernatants after a 24-h stimulation with the native ORF2. This measurement was conducted using commercially available enzyme-linked immunosorbent assay (ELISA) kits, adhering to the manufacturer’s recommendations (R&D Systems, MN, United States).

### 2.9 Evaluation of resistance to gastrointestinal conditions *in vitro*

To evaluate the stability of IBLP027-LysM_5_O2P2 complexes under gastrointestinal conditions, a volume of freshly formed complexes was mixed and incubated at 37°C with an equal volume of buffers mimicking saliva (NaCl 6.2 g/L; KCl 2.2 g/L; CaCl_2_ 0.22 g/L; NaHCO_3_ 1.2 g/L pH 7.2), gastric fluid (NaCl 6.2 g/L; KCl 2.2 g/L; CaCl_2_ 0.22 g/L; NaHCO_3_ 1.2 g/L pH 2.5) or intestinal fluid (NaHCO_3_ 6.4 g/L; KCl 0.239 g/L; NaCl 1.28 g/L; sodium deoxycholate monohydrate 0.5%, pancreatin protease >1,900 USP). Incubations were performed with shaking for 5, 60 and 120 min to simulate digestion in saliva, gastric fluids and intestinal fluids, respectively. Incubation with PBS for 120 min acted as control. The assay was done in duplicate. After each incubation period, samples were examined using SDS-PAGE.

### 2.10 Statistical analysis

Experiments were performed in duplicates and results were expressed as mean ± standard deviation (SD). After verification of the normal distribution of data one-way ANOVA was used. Tukey’s test (for pairwise comparisons of the means) was used to test differences between the groups. Differences were considered significant at *p* < 0.05.

Graphics were prepared using BioRender.

### 2.11 Ethical statement

All methods were carried out in accordance with relevant guidelines and regulations. All experiments were approved by the Ethical Committee of Animal Care CONICET (Research protocol 025/2019).

## 3 Results

### 3.1 Identification of recombinant expression plasmid

A nested PCR product of 552 bp was checked in agarose gel electrophoresis (Supplementary Figure S1A). The entry plasmid pENTR-O2P2 (Supplementary Figure S1B) and the expression plasmid, pENHAc-O2P2 (Supplementary Figure S1C), were confirmed using restriction endonuclease digestion with *BanII* and *EcoRV*, respectively. Agarose gel electrophoresis revealed the backbone matching the expected sizes (Supplementary Figure S1C). The sequencing results aligned with the reference sequences.

### 3.2 Expression and purification of chimeric protein LysM_5_O2P2

We produced a recombinant protein that includes the ORF2 P domain, the most immunogenic domain of HEV-3 ORF2 and five LysM domains, enabling its attachment to the cell wall of immunomodulatory lactobacilli. The recombinant protein, LysM_5_O2P2 (55 kDa), was expressed in *E. coli* Rosetta transformed with pENHAcO2P2. The protein was present mainly in the inclusion bodies which, after being solubilized, kept the peptidoglycan binding properties suggesting a proper refolding of the LysM domains. The protein’s identity was confirmed by SDS-PAGE and western blotting (Supplementary Figure S2). For purification, we captured the chimeric protein from the supernatant to the IBLP surface by its peptidoglycan affinity and subsequently released it using urea. The purified protein was used to generate the IBLP027-LysM_5_O2P2 complexes (Figure 1).

### 3.3 Efficacy of oral IBLP027-LysM_5_O2P2 complexes administration at inducing local but not systemic humoral response

**Figure 3:**
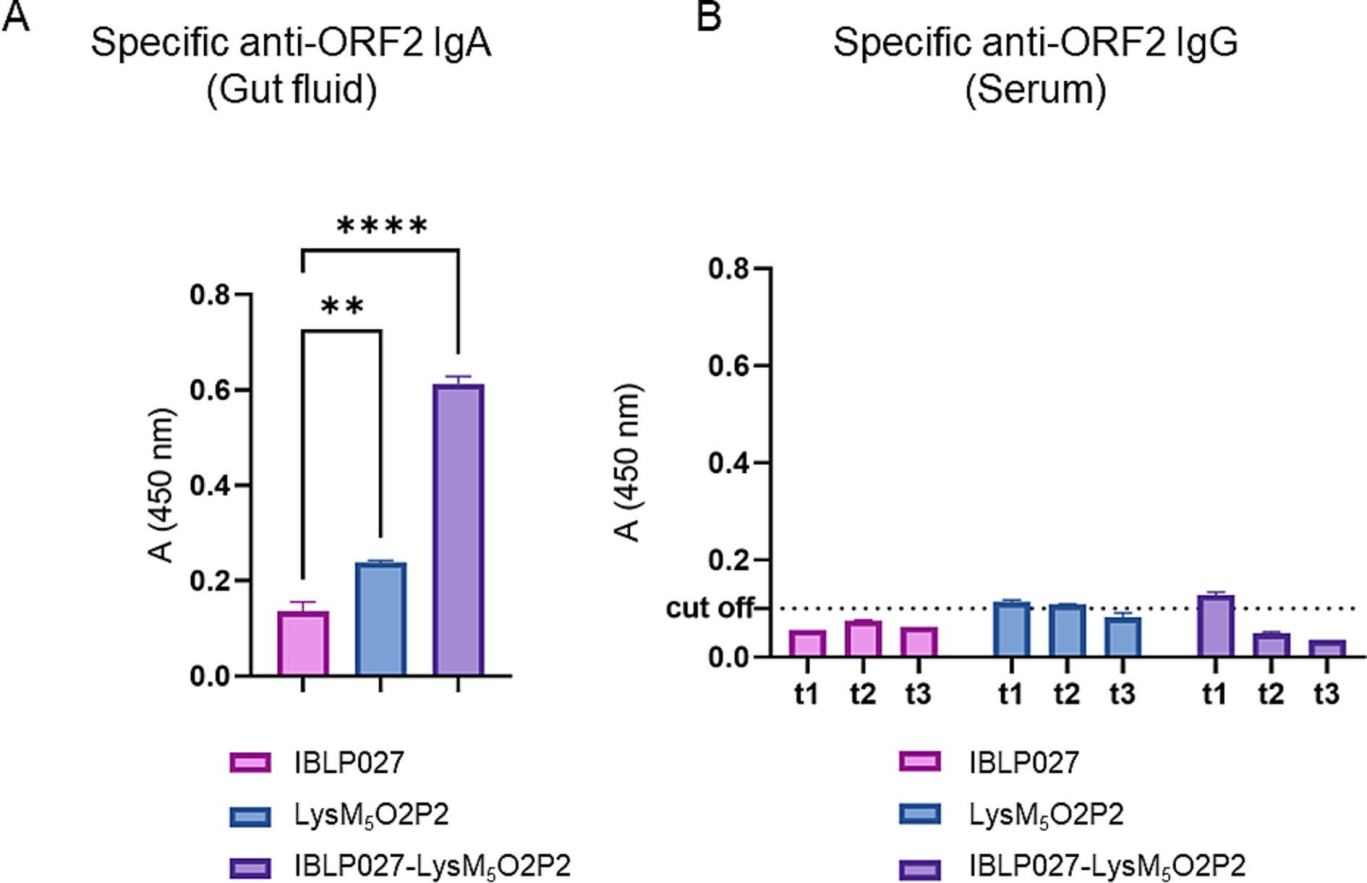
Evaluation of the humoral immune response in mice following the oral immunization schedule. Mice were immunized orally with either IBLP027 (control group), the chimeric protein alone (LysM_5_O2P2) or the IBLP027-LysM_5_O2P2 complexes. **(A)** Specific anti-ORF2 IgA was determined by ELISA in gut fluids obtained from mice at day 35. **(B)** Specific anti-ORF2 IgG was determined by ELISA in sera obtained from mice after 14 (t1), 21 (t2) or 35 (t3) days after the 1st immunization. The cut-off value was set as the mean absorbance ±3 SD from a panel of negative sera (t0). ANOVA was followed by Tukey’s test (for pairwise comparisons of the means) to evaluate the differences between the groups. ^**^p < 0.01, and ^****^p < 0.0001.

To evaluate the immunogenicity elicited by the vaccine prototype, mice received LysM_5_O2P2 alone or displayed on the surface of IBLP027 following a three-dose oral or combined immunization schedule. When following the oral scheme, LysM_5_O2P2 was able to induce specific anti-ORF2 IgA antibodies in the gut, and more importantly, the administration of the complexes induced a significantly (*p* < 0.05) higher level of anti-ORF2 IgA antibodies response as it is shown in Figure 3A, demonstrating once more the adjuvant activity of IBLP027. On the other hand, no specific anti-ORF2 IgG antibodies were detected in the groups immunized with LysM_5_O2P2 or the complexes (Figure 3B).

### 3.4 A combined administration schedule with IBLP027-LysM_5_O2P2 complexes was efficient at inducing mucosal and systemic immune responses

**Figure 4:**
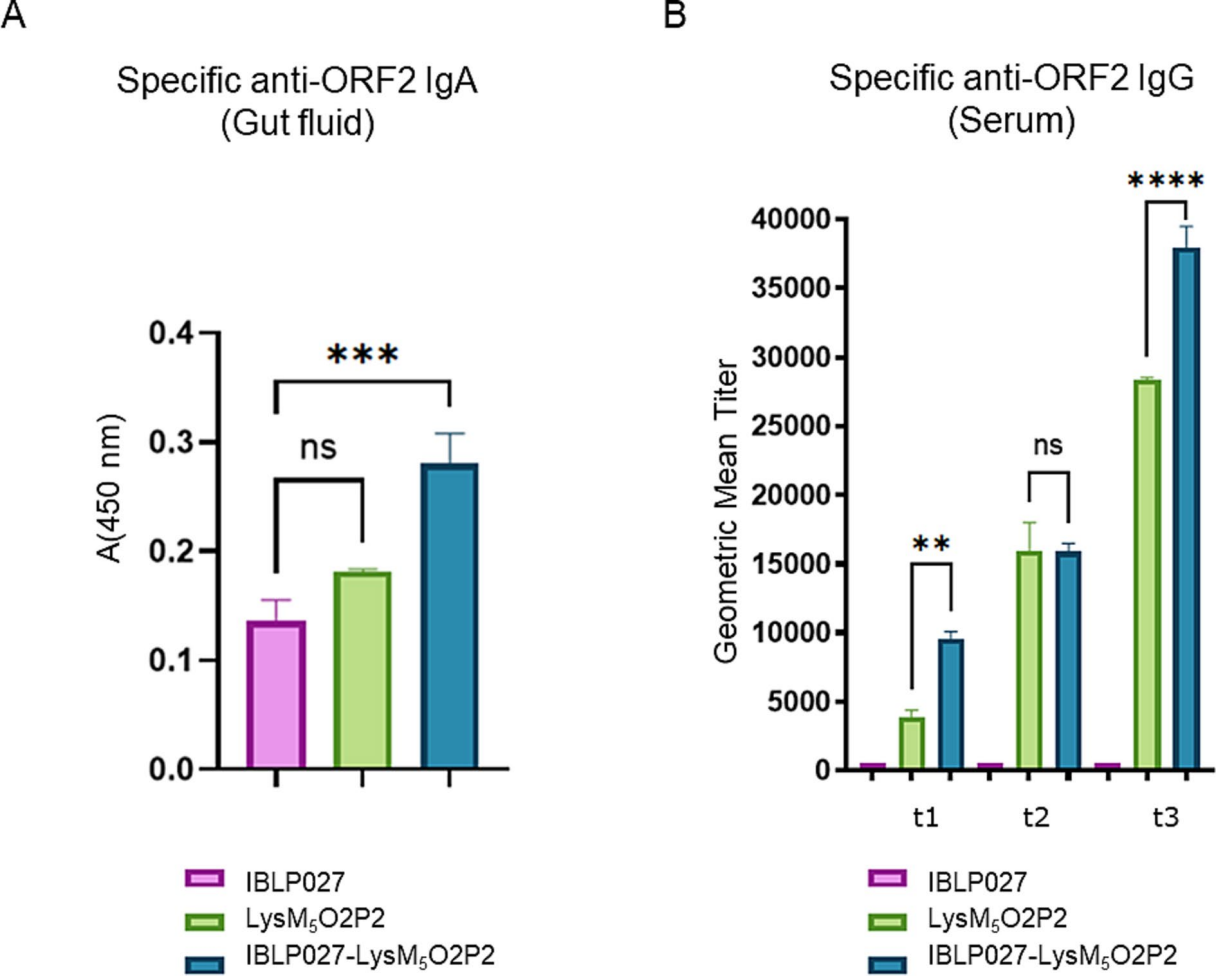
Evaluation of the humoral immune response in mice following the combined immunization schedule. Mice were immunized with a first subcutaneous dose, followed by two nasal boosters with either IBLP027 (control group), the chimeric protein alone (LysM_5_O2P2) or the IBLP027-LysM_5_O2P2 complexes. **(A)** Specific anti-ORF2 IgA was determined by ELISA in gut fluids obtained from mice at day 35. **(B)** Specific anti-ORF2 IgG was determined by ELISA in sera obtained from mice after 14 (t1), 21 (t2) or 35 (t3) days after the 1st immunization. The cut-off value was set as the mean absorbance ±3 SD from a panel of negative sera (t0). ANOVA was followed by Tukey’s test (for pairwise comparisons of the means) to evaluate the differences between the groups. ^***^p < 0.001, and ^****^p < 0.0001.

As it is shown in Figure 4A, when evaluating humoral responses, only the group of mice that were immunized with IBLP027-LysM_5_O2P2 complexes were able to induce specific anti-ORF2 IgA antibodies. Regarding the systemic immune response, after the first dose, the group receiving only LysM_5_O2P2 achieved a geometric mean titer of 3,851 anti-ORF2 antibodies while the mice that were immunized with the complexes elicited a titer of 9,502. The second immunization elicited higher titers anti-ORF2 (15,975 and 15,879), but no significant difference was found between these two groups. When the combined scheme was finished, mice receiving the chimeric protein reached a final titer of 28,354 average while the ones that received LysM_5_O2P2 displayed on the surface of IBLP027 obtained a titer of 37,910 anti-ORF2 antibodies (Figure 4B). Hence, we demonstrated that co-administration with IBLP027 significantly enhances the specific humoral response elicited by LysM_5_O2P2, underscoring the immuno-enhancing effect of IBLP027. Notably, our results indicate that a single subcutaneous dose was enough to initiate a systemic humoral response, with subsequent oral doses further amplifying this response.

**Figure 5:**
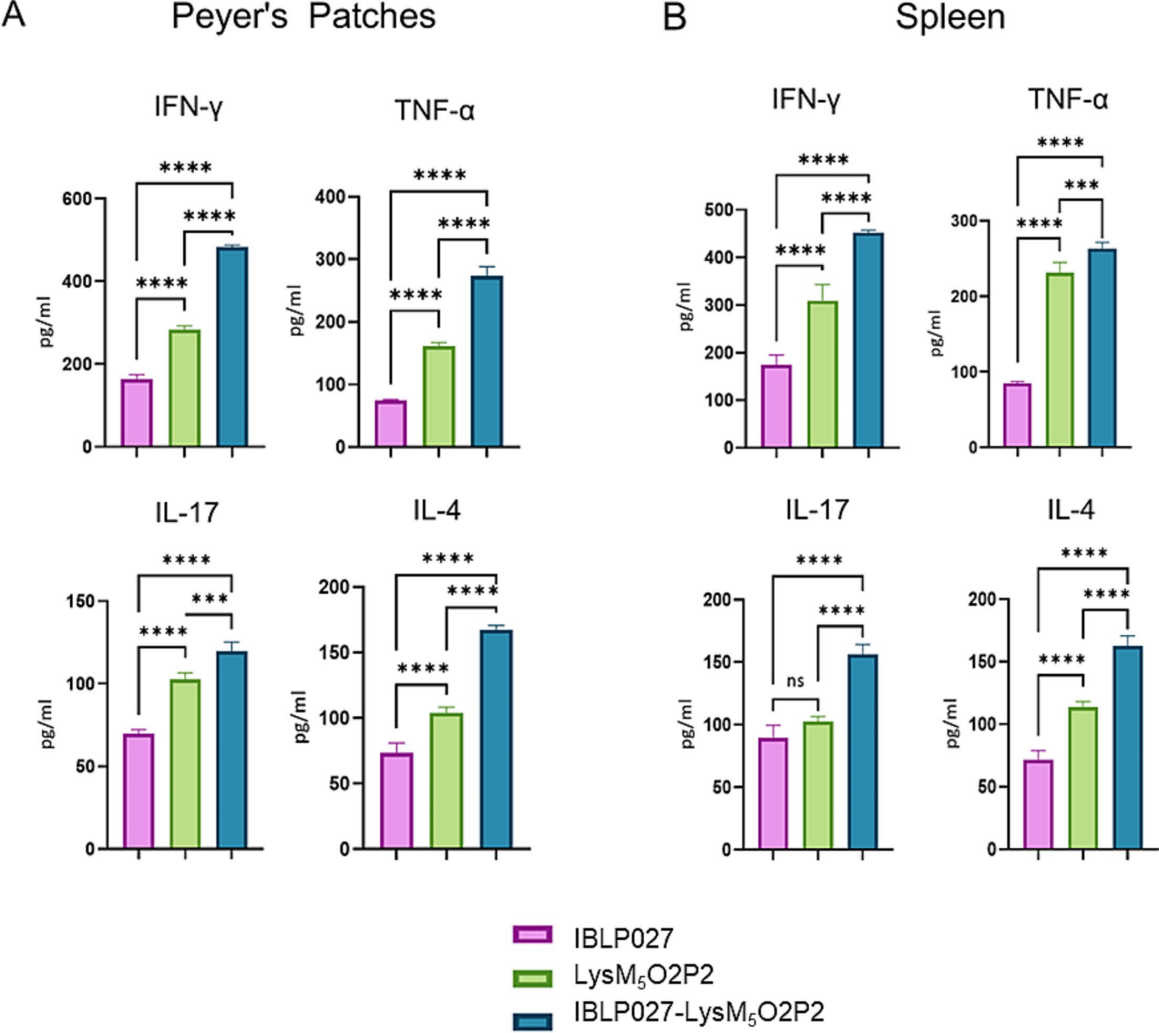
Evaluation of the cellular immune response in mice following the combined immunization schedule. Mice were immunized with a first subcutaneous dose, followed by two nasal boosters with either IBLP027 (control group), the chimeric protein alone (LysM_5_O2P2) or the IBLP027-LysM_5_O2P2 complexes. Peyer’s patches **(A)** or spleens **(B)** were taken at day 35. Cultured immune cells were challenged *ex vivo* with HEV ORF2 and the concentration of TNF-α, IFN-γ, IL-4 and IL-17 were determined in cell culture supernatants after 24 h of antigen stimulation. ANOVA was followed by Tukey’s test (for pairwise comparisons of the means) to evaluate the differences between the groups. ^***^p < 0.001, and ^****^p < 0.0001.

*Ex vivo* stimulation of PP and spleen mononuclear cells with the antigen was performed to assess the specific cellular immune response. Following this stimulation, elevated levels of TNF-α, IFN-γ, and IL-4 were observed in groups immunized with either the chimeric protein or the IBLP027-LysM_5_O2P2 complexes compared to the control group (Figure 5). Notably, the highest concentrations of these cytokines were detected in the group receiving IBLP027-LysM_5_O2P2 complexes. In contrast, enhanced IL-17 responses were observed only in splenocytes obtained from mice immunized with IBLP027-LysM_5_O2P2 complexes (Figure 5B).

### 3.5 LysM_5_O2P2 does not fully resists the gastrointestinal conditions

**Figure 6:**
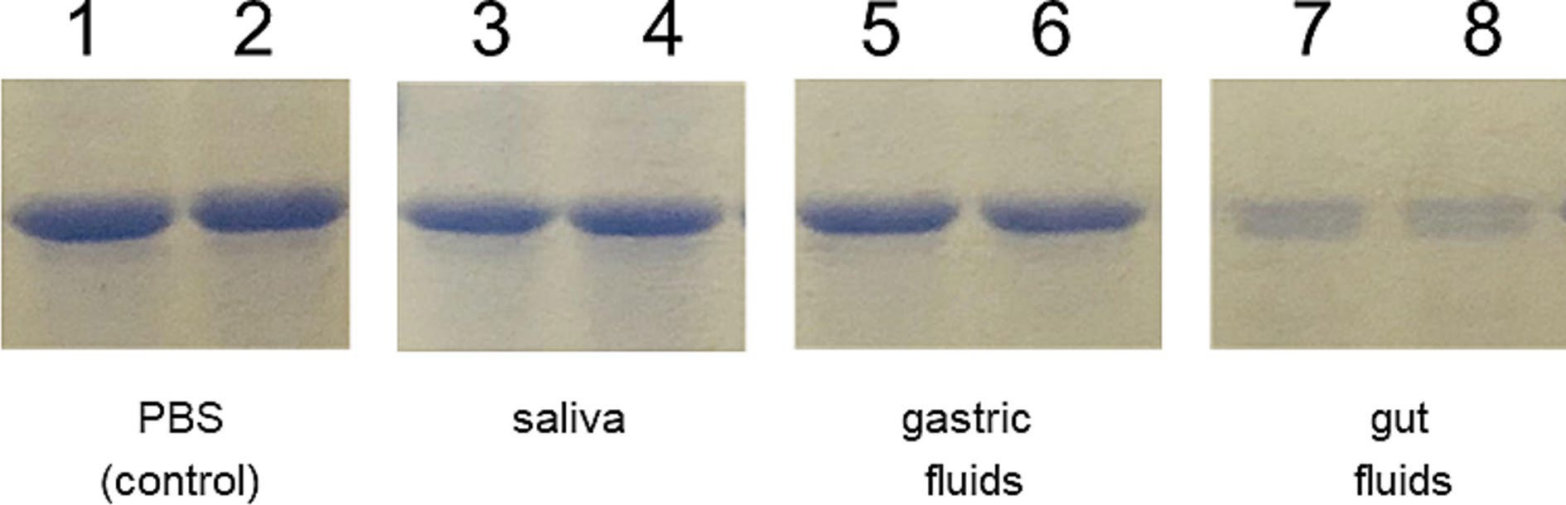
Resistance of the IBLP027-LysM_5_O2P2 complexes to artificial gastrointestinal fluids. After incubating the complexes in the respective buffers that simulate saliva (lines 3–4) for 5 min, gastric fluid for 30 min (lines 5–6) and pancreatic fluid for 90 min (lines 7–8), the complexes were suspended in Lämmli buffer and run in SDS-PAGE. The complexes without treatment served as control (lines 1–2).

Due to the differences observed in the IgG response elicited by the mice following the two immunization schemes, we conducted an *in vitro* gastrointestinal stability assay to assess the integrity of the chimeric protein. The assay involved incubating the IBLP027-LysM_5_O2P2 complexes in buffers simulating saliva, gastric, and intestinal fluids. As shown in Figure 6 the lysozyme in the saliva buffer and the extreme pH of the gastric fluid did not compromise the integrity of the protein attached to the surface of IBLP027. However, the chimeric protein underwent significant proteolysis when exposed to pancreatin in the intestinal fluid. Thus, we hypothesize that during priming, the protein levels were below the threshold needed to trigger an IgG response. This degradation likely explains the absence of IgG antibodies in mice following the oral immunization schedule.

## 4 Discussion

Mucosal vaccines administered orally face numerous challenges, such as digestive enzymes and extreme pH (Kiyono et al., 2021), which must be overcome to deliver antigens to intestinal epithelium to be processed by M cells to reach the antigen presenting cells (APCs) (Snapper, 2018). Additionally, reaching the inductive site with the appropriate concentration is essential for triggering an immune response (Snapper, 2018), since there is a high probability of proteolytic degradation of antigens on mucosal surfaces, may require larger amounts of antigens, potentially risking oral tolerance (Mestecky et al., 2007).

LAB have been widely studied as platforms for mucosal vaccines, including recombinant lactobacilli and lactococci expressing surface antigens. Oral immunization with recombinant *Lactococcus lactis* expressing the protective P protein of *Streptococcus pneumoniae* induced protective immunity in mice, eliciting specific IgG and IgA in gut fluids and increasing IFN-γ and IL-4 production in splenocytes (Villena et al., 2008). Similarly, intranasal delivery of recombinant *L. lactis* expressing the HPV E7 oncoprotein anchored to its cell wall boosted IFN-γ and IL-2 production in splenocytes (Bermúdez-Humarán et al., 2004). These findings highlight LAB’s potential as adjuvants for mucosal vaccines and suggest postbiotic IBLPs as promising platforms for presenting recombinant antigens.

The chimeric protein LysM_5_O2P2 (Supplementary Figures S1, S2) obtained in this work exhibits a strong binding affinity to the peptidoglycan, leading to the formation of IBLP027-LysM_5_O2P2 complexes (Figure 1). Oral immunization with the complexes following either immunization schedule, induced specific anti-ORF2 IgA antibodies (Figures 3A, 4A), which may provide protection at the port of entry. However, beyond a local response, the development of a systemic response is crucial. Indeed, the antibody response in serum represents the most extensively studied immune response to HEV (Walker, 2019; Bryan et al., 1994; Huang et al., 2010). Some studies suggest that reinfections may occur in individuals immunized with low preexisting antibody titers (Bryan et al., 1994), and it has been observed that immunocompromised patients with low IgG levels tend to develop chronic hepatitis (Pas et al., 2012; Dalton et al., 2009). Notably, the oral schedule failed to elicit anti-ORF2 IgG antibodies (Figure 3B), whereas the combined regimen did (Figure 4B). Therefore, the initial subcutaneous dose was necessary to elicit a systemic humoral response, which further induced increasing titers with each subsequent booster dose.

The cellular response was further studied in mice following the combined scheme only. When using PP *ex vivo*, the APCs in the tissue (especially dendritic cells) can still process antigens and present them to T cells, as these cells are already part of this tissue (Yoshida et al., 2002). This model is particularly useful for studying mucosal immune responses. Spleen is a major organ involved in systemic immune responses. Splenocytes, a mix of immune cells including T cells, B cells, and APCs can be isolated, cultured and stimulated with antigens to assess systemic cellular responses. In this study, the *ex vivo* stimulation of primary cultures of splenocytes and mononuclear cells from PP with the antigen ORF2 led to an increased production of IFN-γ, TNF-α, and IL-4 compared to the group immunized with the chimeric protein alone, demonstrating the adjuvant effect of IBLP027 (Figure 5). This suggests that complexes elicit a Th1/Th2 mixed response, consistent with a previous study where oral immunization with recombinant ORF2 protein with either IBLP1505 or IBLP027 resulted in a comparable immune response profile (Arce et al., 2020). Remarkably, IL-17 only increased in mice immunized with the IBLP027-LysM_5_O2P2 complexes (Figure 5). This cytokine influences cell recruitment and plays an important role in establishing long lasting vaccine-induced immunity against viral infections, as shown in a mouse model for rotavirus infection (Lin et al., 2010). Peptidoglycan, the main component of the IBLP, can activate dendritic cells via TLR2 promoting IL-17 production (van Beelen et al., 2007), further supporting IBLP027’s potential as a mucosal adjuvant.

Orally transmitted viruses have evolved to resist the harsh conditions of the host’s gastrointestinal environment, particularly concerning structural proteins. The *in vitro* experiment on gastrointestinal stability showed that a significant amount of LysM_5_O2P2 undergoes proteolysis by intestinal juice enzymes (Figure 6). The ORF2 aa455–602 sequence corresponding to P2 contains 3 trypsin cleavage sites (Wei et al., 2018), typically protected by dimerization. The LysM_5_O2P2 complexes expose the P2 domain on their surfaces. The partial digestion observed in the *in vitro* assay suggests that the trypsin sites are exposed, thus, the protein fails to dimerize, possibly due to the size hindrance of the LysM_5_ anchor or due to the protein being immobilized when bound to the IBLP027 surface. This finding suggests that the partial proteolysis of the LysM_5_O2P2 reduces the final concentration reaching the intestinal epithelium. This may explain why the complexes induce IgA but not systemic IgG in the oral regimen. Predominantly IgA-type humoral response with weak or absent IgG response has been reported in prior studies: in a human trial, intranasal immunization with a measles vaccine in previously immunized individuals elicited a strong IgA response in nasal washes but no serum IgG response was recorded (Simon et al., 2011). One hypothesis is that the vaccine cannot reach the systemic lymphoid tissue, limiting the response to local mucosal antibody production (Simon et al., 2011). Another example is the oral poliovirus vaccine, which predominantly induces local immunity with a strong cellular response and secretory IgA production (Parker and Grassly, 2016; Wahid et al., 2005).

In 2015, the immunogenicity of a recombinant strain of *L. lactis* expressing the C-terminal of ORF2 (spanning 459–606 aa) on its surface was evaluated (Gao et al., 2015). Mice were immunized with three oral doses, which induced a specific mucosal humoral response comparable to that observed in our study using the IBLP027-LysM_5_O2P2 complexes, regardless of the immunization schedule (Figures 3A, 4A). However, unlike the findings reported by Gao et al. (2015), which reported a slight IgG response, mice immunized with the IBLP027-LysM_5_O2P2 complexes via the oral route only did not exhibit a detectable IgG response (Figure 3B). In contrast, mice immunized using the combined regimen showed progressively increasing specific IgG serum titers with each immunization (Figure 4B). Regarding the cellular response, *ex vivo* stimulation of splenocytes from mice immunized with *L. lactis* expressing ORF2 led to increased IL-4 production, with no significant changes in IFN-γ levels indicating a Th2-skewed immune response (Gao et al., 2015). Notably, our results demonstrated that administering IBLP027-LysM_5_O2P2 complexes under the combined regimen induced elevated production of both IL-4 and IFN-γ, consistent with a more desirable mixed Th1/Th2 response (Figure 5).

The combination of subcutaneous and oral vaccines effectively induces both systemic and mucosal immune responses. Evidence suggests that mucosal vaccines as boosters after parenteral priming enhance protection against reinfections. For example, in rats, oral boosts following subcutaneous SARS-CoV-2 vaccination significantly increased neutralizing antibody levels, with one oral boost achieving serum IgG and mucosal IgA levels comparable to three subcutaneous doses (Pitcovski et al., 2022). Similarly, an oral vaccine followed by a subcutaneous boost using a *Helicobacter pylori* fusion protein enhanced serum IgG levels, specific IgG subtypes, and CD4^+^ T cell responses, including IFN-γ and IL-17A secretion (Zhang et al., 2022).

In conclusion, we developed a promising subunit vaccine candidate against HEV using IBLP027 as both a carrier and adjuvant. The combined immunization regimen with IBLP027-LysM5O2P2 complexes successfully induced systemic and mucosal immune responses. The vaccine elicited specific IgA for mucosal immunity, crucial at the site of viral entry, and IgG for systemic protection, comparable to the commercial Hecolin^®^ vaccine. This dual immune activation makes the vaccine a compelling candidate, especially for swine vaccination targeting a major zoonotic reservoir and with the potential of reducing HEV transmission risk. Animal HEV infection models are limited, especially because mice are not susceptible to infection and models like swine are costly to maintain and challenging to handle. Nonetheless, a virus infection trial is necessary to confirm the potential of the proposed experimental vaccine and the combined administration schedule.

## Data Availability

The raw data supporting the conclusions of this article will be made available by the authors, without undue reservation.

## Supplementary material

SUPPLEMENTARY FIGURE S1 Construction of recombinant plasmids. **(A)** The O2P2 PCR product was obtained from a nested-PCR where the sequence corresponding to ORF2 GT3 aa 452–608 was amplified with the corresponding *att* sites. **(B)** Enzymatic restriction of pENTRO2P2 plasmid with *BanII*. **(C)** The expression plasmid pENHAcO2P2 was constructed after a LR recombination. The empty destination plasmid pENHAc[rfB] and the constructed pENHAcO2P2 were digested by EcoRV and checked by agarose electrophoresis.

SUPPLEMENTARY FIGURE S2 SDS-PAGE and western blot. Expression, purification and confirmation of LysM5O2P2 identity by **(A)** SDS-PAGE followed by Coomassie staining. Line 1: Molecular marker, line 2: purified chimeric LysM5O2P2 protein obtained by eluting the protein from the complexes using 8 M urea. **(B)** Western blotting using mouse anti-His as a primary antibody and anti-mouse IgG labeled with horseradish peroxidase as secondary antibody. Lines 2 and 3: Complexes with a low and high amount of bound chimeric protein, respectively. Arrow heads indicate the band corresponding to O2P2.

## References

[ref1] Arce L. P. Raya Tonetti M. F. Raimondo M. P. Müller M. F. Salva S. Álvarez S.. (2020). Oral vaccination with hepatitis E virus capsid protein and immunobiotic bacterium-like particles induce intestinal and systemic immunity in mice. Probiotics Antimicrob. Proteins 12, 961–972. doi: 10.1007/s12602-019-09598-7, PMID: 31630331

[ref2] Bermúdez-Humarán L. G. Cortes-Perez N. G. Le Loir Y. Alcocer-González J. M. Tamez-Guerra R. S. de Oca-Luna R. M.. (2004). An inducible surface presentation system improves cellular immunity against human papillomavirus type 16 E7 antigen in mice after nasal administration with recombinant lactococci. J. Med. Microbiol. 53, 427–433. doi: 10.1099/jmm.0.05472-0, PMID: 15096553

[ref3] Bosma T. Kanninga R. Neef J. Audouy S. A. L. van Roosmalen M. L. Steen A.. (2006). Novel surface display system for proteins on non-genetically modified gram-positive bacteria. Appl. Environ. Microbiol. 72, 880–889. doi: 10.1128/AEM.72.1.880-889.2006, PMID: 16391130 PMC1352190

[ref4] Bryan J. P. Tsarev S. A. Iqbal M. Ticehurst J. Emerson S. Ahmed A.. (1994). Epidemic hepatitis E in Pakistan: patterns of serologic response and evidence that antibody to hepatitis E virus protects against disease. J. Infect. Dis. 170, 517–521. doi: 10.1093/infdis/170.3.517, PMID: 8077708

[ref5] Cancela F. Noceti O. Arbiza J. Mirazo S. (2022). Structural aspects of hepatitis E virus. Arch. Virol. 167, 2457–2481. doi: 10.1007/s00705-022-05575-8, PMID: 36098802 PMC9469829

[ref6] Dalton H. R. Bendall R. P. Keane F. E. Tedder R. S. Ijaz S. (2009). Persistent carriage of hepatitis E virus in patients with HIV infection. N. Engl. J. Med. 361, 1025–1027. doi: 10.1056/NEJMc0903778, PMID: 19726781

[ref7] Desvaux M. Dumas E. Chafsey I. Hébraud M. (2006). Protein cell surface display in gram-positive bacteria: from single protein to macromolecular protein structure. FEMS Microbiol. Lett. 256, 1–15. doi: 10.1111/j.1574-6968.2006.00122.x, PMID: 16487313

[ref8] Fabrizi F. Cerutti R. Garcia-Agudo R. Bellincioni C. Porata G. Frontini G.. (2020). Adjuvanted recombinant HBV vaccine (HBV-AS04) is effective over extended follow-up in dialysis population. An open-label non randomized trial. Clin. Res. Hepatol. Gastroenterol. 44, 905–912. doi: 10.1016/j.clinre.2020.01.010, PMID: 32144074

[ref9] Gao S. Li D. Liu Y. Zha E. Zhou T. Yue X. (2015). Oral immunization with recombinant hepatitis E virus antigen displayed on the *Lactococcus lactis* surface enhances ORF2-specific mucosal and systemic immune responses in mice. Int. Immunopharmacol. 24, 140–145. doi: 10.1016/j.intimp.2014.10.032, PMID: 25445956

[ref10] Huang S. Zhang X. Jiang H. Yan Q. Ai X. Wang Y.. (2010). Profile of acute infectious markers in sporadic hepatitis E. PLoS One 5:e13560. doi: 10.1371/journal.pone.0013560, PMID: 21042408 PMC2958841

[ref11] Huang S. Zhang X. Su Y. Zhuang C. Tang Z. Huang X.. (2024). Long-term efficacy of a recombinant hepatitis E vaccine in adults: 10-year results from a randomised, double-blind, placebo-controlled, phase 3 trial. Lancet 403, 813–823. doi: 10.1016/S0140-6736(23)02234-1, PMID: 38387470

[ref12] Kao C. M. Rostad C. A. Nolan L. E. Peters E. Kleinhenz J. Sherman J. D.. (2024). A phase 1, double-blinded, placebo-controlled clinical trial to evaluate the safety and immunogenicity of HEV-239 (Hecolin^®^) vaccine in healthy US adults. J. Infect. Dis. 230, 1093–1101. doi: 10.1093/infdis/jiae148, PMID: 38536442 PMC11565884

[ref13] Kiyono H. Yuki Y. Nakahashi-Ouchida R. Fujihashi K. (2021). Mucosal vaccines: wisdom from now and then. Int. Immunol. 33, 767–774. doi: 10.1093/intimm/dxab056, PMID: 34436595 PMC8633596

[ref14] Lenart K. Arcoverde Cerveira R. Hellgren F. Ols S. Sheward D. J. Kim C.. (2024). Three immunizations with Novavax’s protein vaccines increase antibody breadth and provide durable protection from SARS-CoV-2. npj Vaccines 9:17. doi: 10.1038/s41541-024-00806-2, PMID: 38245545 PMC10799869

[ref15] Li E. Chi H. Huang P. Yan F. Zhang Y. Liu C.. (2019). A novel bacterium-like particle vaccine displaying the MERS-CoV receptor-binding domain induces specific mucosal and systemic immune responses in mice. Viruses 11:799. doi: 10.3390/v11090799, PMID: 31470645 PMC6784119

[ref16] Lin Y. Slight S. R. Khader S. A. (2010). Th17 cytokines and vaccine-induced immunity. Semin. Immunopathol. 32, 79–90. doi: 10.1007/s00281-009-0191-2, PMID: 20112107 PMC2855296

[ref17] Liu W. Tan Z. Liu H. Zeng Z. Luo S. Yang H.. (2017). Nongenetically modified *Lactococcus lactis*-adjuvanted vaccination enhanced innate immunity against *Helicobacter pylori*. Helicobacter 22:e12426. doi: 10.1111/hel.12426, PMID: 28805287

[ref18] Marion O. Capelli N. Lhomme S. Dubois M. Pucelle M. Abravanel F.. (2019). Hepatitis E virus genotype 3 and capsid protein in the blood and urine of immunocompromised patients. J. Infect. 78, 232–240. doi: 10.1016/j.jinf.2019.01.004, PMID: 30659856

[ref19] Mestecky J. Russell M. W. Elson C. O. (2007). Perspectives on mucosal vaccines: is mucosal tolerance a barrier? J. Immunol. 179, 5633–5638. doi: 10.4049/jimmunol.179.9.5633 17947632

[ref20] Michon C. Langella P. Eijsink V. G. H. Mathiesen G. Chatel J. M. (2016). Display of recombinant proteins at the surface of lactic acid bacteria: strategies and applications. Microb. Cell Fact. 15:70. doi: 10.1186/s12934-016-0468-9, PMID: 27142045 PMC4855500

[ref21] Nesbitt R. C. Asilaza V. K. Gignoux E. Koyuncu A. Gitahi P. Nkemenang P.. (2024). Vaccination coverage and adverse events following a reactive vaccination campaign against hepatitis E in Bentiu displaced persons camp, South Sudan. PLoS Negl. Trop. Dis. 18:e0011661. doi: 10.1371/journal.pntd.0011661, PMID: 38252655 PMC10833508

[ref22] Øverbø J. Aziz A. Zaman K. Clemens J. Halle Julin C. Qadri F.. (2023). Immunogenicity and safety of a two-dose regimen with hepatitis E virus vaccine in healthy adults in rural Bangladesh: a randomized, double-blind, controlled, phase 2/pilot trial. Vaccine 41, 1059–1066. doi: 10.1016/j.vaccine.2022.12.064, PMID: 36599736

[ref23] Parker E. P. K. Grassly N. C. (2016). Unravelling mucosal immunity to poliovirus. Lancet Infect. Dis. 16, 1310–1311. doi: 10.1016/S1473-3099(16)30371-1, PMID: 27638358

[ref24] Pas S. D. de Man R. A. Mulders C. Balk A. H. M. M. van Hal P. T. W. Weimar W.. (2012). Hepatitis E virus infection among solid organ transplant recipients, the Netherlands. Emerg. Infect. Dis. 18, 869–872. doi: 10.3201/eid1805.111712, PMID: 22516170 PMC3358074

[ref25] Pitcovski J. Gruzdev N. Abzach A. Katz C. Ben-Adiva R. Brand-Shwartz M.. (2022). Oral subunit SARS-CoV-2 vaccine induces systemic neutralizing IgG, IgA and cellular immune responses and can boost neutralizing antibody responses primed by an injected vaccine. Vaccine 40, 1098–1107. doi: 10.1016/j.vaccine.2022.01.025, PMID: 35078662 PMC8768024

[ref26] Raya Tonetti F. Arce L. Salva S. Alvarez S. Takahashi H. Kitazawa H.. (2020). Immunomodulatory properties of bacterium-like particles obtained from Immunobiotic lactobacilli: prospects for their use as mucosal adjuvants. Front. Immunol. 11:15. doi: 10.3389/fimmu.2020.00015, PMID: 32038659 PMC6989447

[ref27] Raya-Tonetti F. Müller M. Sacur J. Kitazawa H. Villena J. Vizoso-Pinto M. G. (2021). Novel LysM motifs for antigen display on lactobacilli for mucosal immunization. Sci. Rep. 11:21691. doi: 10.1038/s41598-021-01087-8, PMID: 34737363 PMC8568972

[ref28] Sayed I. M. Meuleman P. (2020). Updates in hepatitis E virus (HEV) field; lessons learned from human liver chimeric mice. Rev. Med. Virol. 30:e2086. doi: 10.1002/rmv.2086, PMID: 31835277

[ref29] Sharma S. Kumar A. Kar P. Agarwal S. Ramji S. Husain S. A.. (2017). Risk factors for vertical transmission of hepatitis E virus infection. J. Viral Hepat. 24, 1067–1075. doi: 10.1111/jvh.12730, PMID: 28570034

[ref30] Simon J. K. Ramirez K. Cuberos L. Campbell J. D. Viret J. F. Muñoz A.. (2011). Mucosal IgA responses in healthy adult volunteers following intranasal spray delivery of a live attenuated measles vaccine. Clin. Vaccine Immunol. 18, 355–361. doi: 10.1128/CVI.00354-10, PMID: 21228137 PMC3067370

[ref31] Snapper C. M. (2018). Distinct immunologic properties of soluble versus particulate antigens. Front. Immunol. 9:598. doi: 10.3389/fimmu.2018.00598, PMID: 29619034 PMC5871672

[ref32] Strategic Advisory Group of Experts. (2024). Hepatitis E vaccines background paper Strategic Advisory Group of Experts (SAGE) on Immunization meeting. Available at: https://cdn.who.int/media/docs/default-source/immunization/sage/2024/march/hepatitis_e_background_paper_sage_mar24.pdf?sfvrsn=e14438c4_1 (Accessed March 12, 2024). 10.3390/vaccines12121402 PMC11680103 39772062

[ref33] Sudo H. Tokunoh N. Tsujii A. Kawashima S. Hayakawa Y. Fukushima H.. (2023). The adjuvant effect of bacterium-like particles depends on the route of administration. Front. Immunol. 14:1082273. doi: 10.3389/fimmu.2023.1082273, PMID: 36742329 PMC9892444

[ref34] van Beelen A. J. Zelinkova Z. Taanman-Kueter E. W. Muller F. J. Hommes D. W. Zaat S. A. J.. (2007). Stimulation of the intracellular bacterial sensor NOD2 programs dendritic cells to promote interleukin-17 production in human memory T cells. Immunity 27, 660–669. doi: 10.1016/j.immuni.2007.08.013, PMID: 17919942

[ref35] Villena J. Kitazawa H. (2020). The modulation of mucosal antiviral immunity by immunobiotics: could they offer any benefit in the SARS-CoV-2 pandemic? Front. Physiol. 11:699. doi: 10.3389/fphys.2020.00699, PMID: 32670091 PMC7326040

[ref36] Villena J. Medina M. Raya R. Alvarez S. (2008). Oral immunization with recombinant *Lactococcus lactis* confers protection against respiratory pneumococcal infection. Can. J. Microbiol. 54, 845–853. doi: 10.1139/W08-077, PMID: 18923553

[ref37] Vipond C. Sutherland J. Nordgren K. Kemp G. Heath A. Care R.. (2019). Development and validation of a monocyte activation test for the control/safety testing of an OMV-based meningococcal B vaccine. Vaccine 37, 3747–3753. doi: 10.1016/j.vaccine.2018.06.038, PMID: 31202503

[ref38] Vizoso Pinto M. G. Pfrepper K.-I. Janke T. Noelting C. Sander M. Lueking A.. (2010). A systematic approach for the identification of novel, serologically reactive recombinant Varicella-Zoster Virus (VZV) antigens. Virol. J. 7:165. doi: 10.1186/1743-422X-7-165, PMID: 20646309 PMC2915977

[ref39] Wahid R. Cannon M. J. Chow M. (2005). Virus-specific CD4^+^ and CD8^+^ cytotoxic T-cell responses and long-term T-cell memory in individuals vaccinated against polio. J. Virol. 79, 5988–5995. doi: 10.1128/JVI.79.10.5988-5995.2005, PMID: 15857985 PMC1091702

[ref40] Walker C. M. (2019). Adaptive immune responses in hepatitis a virus and hepatitis E virus infections. Cold Spring Harb. Perspect. Med. 9:a033472. doi: 10.1101/cshperspect.a033472, PMID: 29844218 PMC6531370

[ref41] Wang H. Li P. Zhang M. Bi J. He Y. Li F.. (2022). Vaccine with bacterium-like particles displaying HIV-1 gp120 trimer elicits specific mucosal responses and neutralizing antibodies in rhesus macaques. Microb. Biotechnol. 15, 2022–2039. doi: 10.1111/1751-7915.14022, PMID: 35290714 PMC9249329

[ref42] Wang L. Liu L. Wang L. (2018). An overview: rabbit hepatitis E virus (HEV) and rabbit providing an animal model for HEV study. Rev. Med. Virol. 28:e1961. doi: 10.1002/rmv.1961, PMID: 29148605

[ref43] Webb G. W. Dalton H. R. (2019). Hepatitis E: an underestimated emerging threat. Ther. Adv. Infect. Dis. 6:204993611983716. doi: 10.1177/2049936119837162, PMID: 30984394 PMC6448100

[ref44] Wei W. Behloul N. Baha S. Liu Z. Aslam M. S. Meng J. (2018). Dimerization: a structural feature for the protection of hepatitis E virus capsid protein against trypsinization. Sci. Rep. 8:1738. doi: 10.1038/s41598-018-20137-2, PMID: 29379064 PMC5788867

[ref45] World Health Organization. (2015). Hepatitis E vaccine: WHO position paper, May 2015. Available at: https://iris.who.int/bitstream/handle/10665/242352/WER9018_185-200.PDF?sequence=1 (Accessed March 12, 2024).

[ref46] World Health Organization. (2023). Hepatitis E: fact sheet. Available at: http://www.who.int/mediacentre/factsheets/fs280/en/ (Accessed March 12, 2024).

[ref47] Wu C. Wu X. Xia J. (2020). Hepatitis E virus infection during pregnancy. Virol. J. 17:73. doi: 10.1186/s12985-020-01343-9, PMID: 32522266 PMC7286216

[ref48] Yin X. Feng Z. (2019). Hepatitis E virus entry. Viruses 11:883. doi: 10.3390/v11100883, PMID: 31547135 PMC6832200

[ref49] Yoshida T. Hachimura S. Ishimori M. Kinugasa F. Ise W. Totsuka M.. (2002). Antigen presentation by Peyer’s patch cells can induce both Th1-and Th2-type responses depending on antigen dosage, but a different cytokine response pattern from that of spleen cells. Biosci. Biotechnol. Biochem. 66, 963–969. doi: 10.1271/bbb.66.963, PMID: 12092847

[ref50] Zaman K. Schuind A. E. Adjei S. Antony K. Aponte J. J. Buabeng P. B.. (2024). Safety and immunogenicity of Innovax bivalent human papillomavirus vaccine in girls 9-14 years of age: interim analysis from a phase 3 clinical trial. Vaccine 42, 2290–2298. doi: 10.1016/j.vaccine.2024.02.077, PMID: 38431444 PMC11007388

[ref51] Zhang X. Sang S. Guan Q. Tao H. Wang Y. Liu C. (2022). Oral administration of a *Shigella* 2aT32-based vaccine expressing Ure B-HspA fusion antigen with and without parenteral rUreB-HspA boost confers protection against *Helicobacter pylori* in mice model. Front. Immunol. 13:894206. doi: 10.3389/fimmu.2022.894206, PMID: 35769459 PMC9234132

[ref52] Zhang X. Wei M. Sun G. Wang X. Li M. Lin Z.. (2016). Real-time stability of a hepatitis E vaccine (Hecolin^®^) demonstrated with potency assays and multifaceted physicochemical methods. Vaccine 34, 5871–5877. doi: 10.1016/j.vaccine.2016.10.045, PMID: 27793484

[ref53] Zhao M. Li X.-J. Tang Z.-M. Yang F. Wang S.-L. Cai W.. (2015). A comprehensive study of neutralizing antigenic sites on the hepatitis E virus (HEV) capsid by constructing, clustering, and characterizing a tool box. J. Biol. Chem. 290, 19910–19922. doi: 10.1074/jbc.M115.649764, PMID: 26085097 PMC4528150

