# Supplementary figures and images for: An experimental chimeric hepatitis E virus vaccine elicits both local and systemic immune responses
Source: Front Microbiol. 2024 Dec 24;15:1512018. doi: 10.3389/fmicb.2024.1512018 (PMC11704494; doi:10.3389/fmicb.2024.1512018)

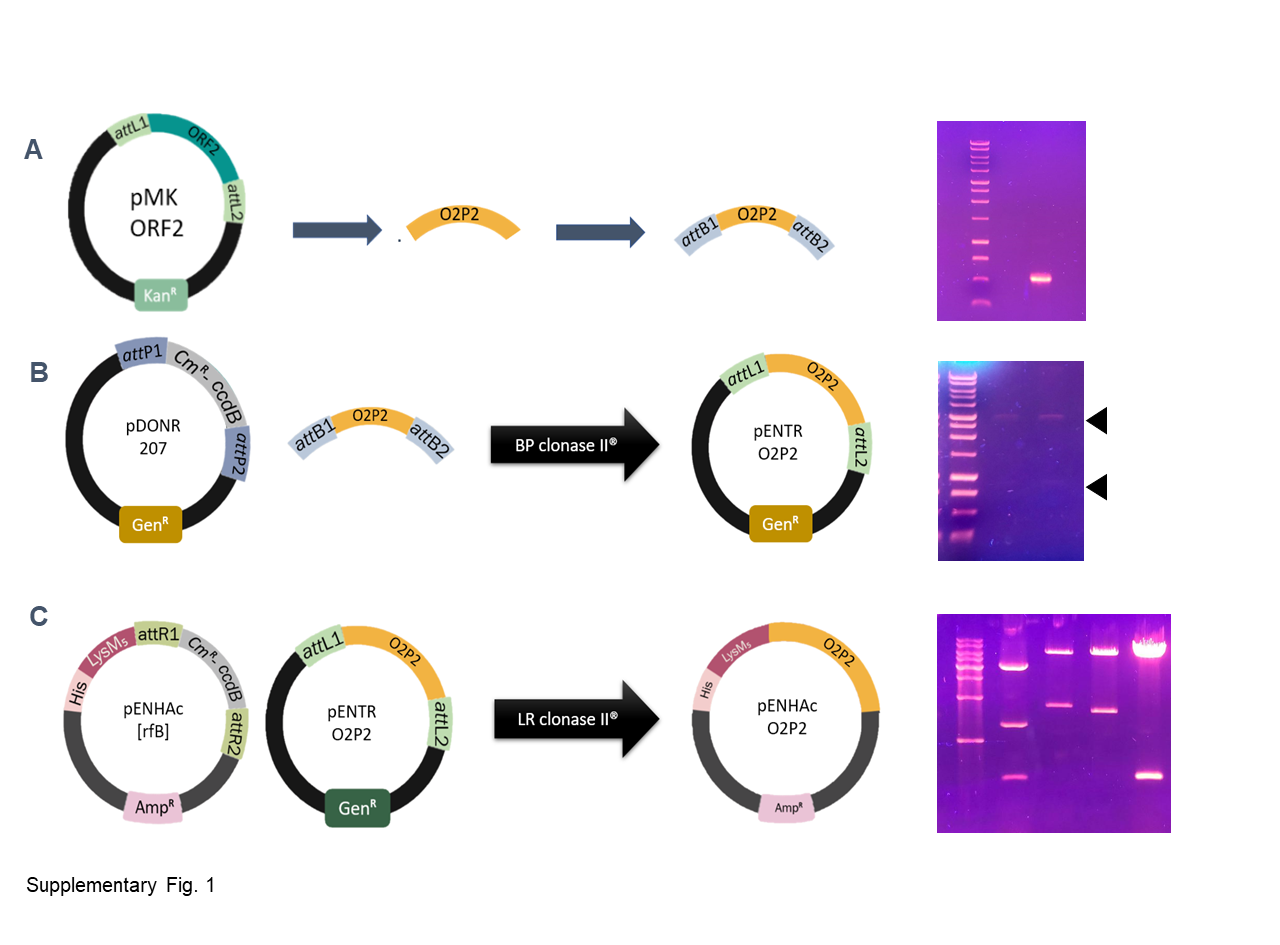

Supplement: SUPPLEMENTARY FIGURE S1 — Construction of recombinant plasmids. (A) The O2P2 PCR product was obtained from a nested-PCR where the sequence corresponding to ORF2 GT3 aa 452–608 was amplified with the corresponding att sites. (B) Enzymatic restriction of pENTRO2P2 plasmid with BanII. (C) The expression plasmid pENHAcO2P2 was constructed after a LR recombination. The empty destination plasmid pENHAc[rfB] and the constructed pENHAcO2P2 were digested by EcoRV and checked by agarose electrophoresis. [file Image_1.TIF]

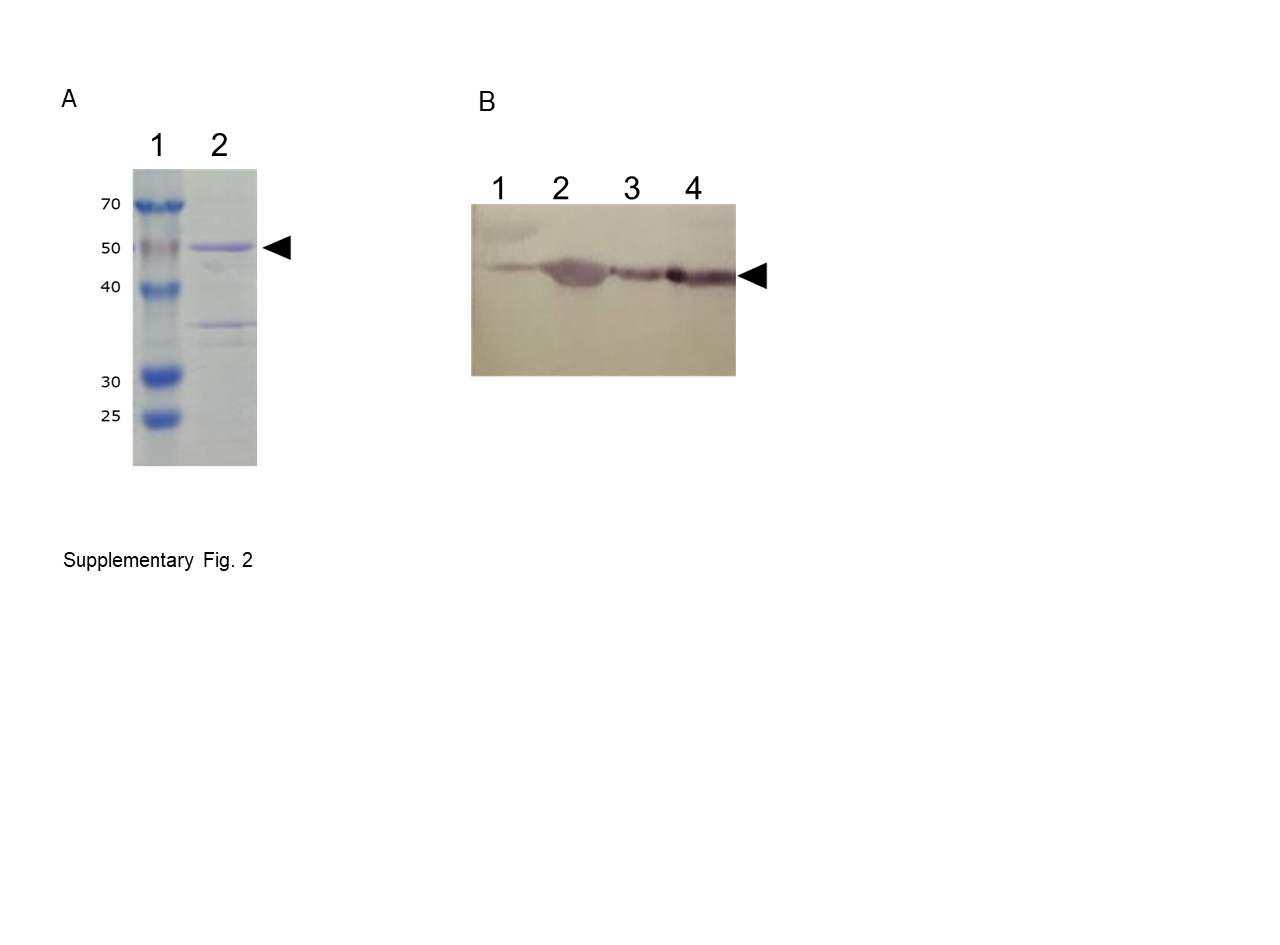

Supplement: SUPPLEMENTARY FIGURE S2 — SDS-PAGE and western blot. Expression, purification and confirmation of LysM5O2P2 identity by (A) SDS-PAGE followed by Coomassie staining. Line 1: Molecular marker, line 2: purified chimeric LysM5O2P2 protein obtained by eluting the protein from the complexes using 8 M urea. (B) Western blotting using mouse anti-His as a primary antibody and anti-mouse IgG labeled with horseradish peroxidase as secondary antibody. Lines 2 and 3: Complexes with a low and high amount of bound chimeric protein, respectively. Arrow heads indicate the band corresponding to O2P2. [file Image_2.TIF]
